# A search for the common ground between Tic; Obsessive-compulsive and Autism Spectrum Disorders: part I, Tic disorders

**DOI:** 10.3934/genet.2017.1.32

**Published:** 2017-02-21

**Authors:** Jarrett Barnhill, James Bedford, James Crowley, Takahiro Soda

**Affiliations:** 1 Department of Psychiatry, University of North Carolina School of Medicine, Chapel Hill, NC, USA; 2 Department of Genetics, University of North Carolina, Chapel Hill, NC, USA; 3 Department of Clinical Neuroscience, Karolinska Institutet, Stockholm, Sweden

**Keywords:** Tics, movement disorders, neurobiology, genetics, endophenotypes

## Abstract

This article is the first of four articles designed to explore the complex interrelationship between Autism Spectrum Disorders (ASD); Obsessive compulsive and Related Disorders (OCRD) and Tic Disorders/Tourette's Syndrome (TD/TS). We begin with an overview TD/TS and follow-up with reviews of OCRD and ASD. The final article in this series represents a synthesis of the neurobiological and genetic markers shared by patients presenting with all three syndromes. The goal is to describe the complex endophenotype of these patients in an effort to better define gene markers that underlie these heterogeneous clinical syndromes. Tic disorders (TD) are a collection of hyperkinetic movements that begin in early childhood. Tics are transient for most affected preschool children but a subgroup development persistent movements or progress to develop Tourette Syndrome (TS). TDs as a group display high heritability rates but definitive gene markers still elude us. The difficulty defining genetic markers is in large part due to the diverse neurodevelopmental trajectory, changing topography and typology, development of a broad spectrum of neurocognitive and behavioral complications, and a mixed pattern of psychiatric comorbidities.

## 1. Introduction

This paper is the first in a series designed to explore the complex genetic relationships between Tic Disorders (TD), Obsessive-compulsive and Related Disorders (OCRD), Intellectual Disability (ID or Intellectual Developmental Disorder), and Autism Spectrum Disorders (ASD). The focus of this initial paper is on Tic Disorders (TD)/Tourette Syndrome (TS). Our investigation begins with Tic Disorders as a hyperkinetic movement disorder but then progresses to TS as a more complex neuropsychiatric syndrome associated with multiple, comorbid psychiatric and neurodevelopmental disorders. This level of complexity is in large part related to the tendency of both tics and associated clinical symptoms to evolve along a continuum that moves from largely involuntary movements to a more complex mixture of sensorimotor, affective, behavioral, neurocognitive and neuropsychiatric symptoms.

The goal of this paper is to present TD/TS in terms of its diverse phenomenology, neurobiology and complex genetics. Subsequent articles in this series will apply this methodology to Autism Spectrum Disorder (ASD) and Obsessive-compulsive and Related Disorders (OCRD). The final article in this series will concentrate on integrating and synthesizing the complex genetics and neurobiology of combinations that includes TD + OCD; TD + ASD; OCD + ASD, and ASD + TD + OCD. The authors selected this approach to better define a set of neurobiological endophenotypes that will enhance our understanding of the core features of these heterogeneous disorders. Although we will not formally discuss Intellectual Disability (Intellectual Developmental Disorder) in our formulation, we acknowledge the critical role of ID (IDD) in shaping the severity of ASD; contributing to self-injurious behavior (SIB), aggression and stereotypies, and the clinical expression of TD/TS and OCRD [1]–[6].

## 2. Tic disorders

Tics are sudden, intrusive, rapid, recurrent, non-rhythmic movements or vocalizations that seem to have no implicit goal or function. Most tics are involuntary and, like other hyperkinetic movement disorders, tend to wax and wane in intensity, worsen during periods of physiological and psychological stress, but improve or disappear during sleep. As a rule, early onset tics begin during the preschool period, especially among children with pre-existing developmental disorders [7]–[11]. Most descriptions of tics focus primarily on them as simple involuntary vocalizations or motor tics but do not delve into their complex diversity. Much of this variability is due to factors related to age of onset, gender (2–4 males are affected for every female), changing typology, severity and complexity of the tics, and the presence of neuropsychiatric comorbidity.

Tic disorders are classified among the Neurodevelopmental Disorders in the DSM-5. The criteria for diagnosing TD requires not only the presence of abnormal movements and vocalizations, but also includes duration and intensity criteria. Symptoms must be sufficient to contribute to functional impairment in social, emotional, and academic or executive skills. Tics should be present by age 18 (or during the developmental period) but in many cases, mild tics may be overlooked. The problem of recognition is compounded by the presence of ID and ASD for reasons that will be explored later. For now, it is sufficient to state that among patients with ASD and ID the age of onset may be less relevant than the age of recognition.

Most children who present with tics during their preschool years generally become tic-free within several months. They are rarely diagnosed but would generally fall into the category Other Specified and Unspecified Tic disorders. A smaller percentage of children develop persistent tics that extend beyond 3 months. They are usually diagnosed as either Provisional Tic Disorder, or Tic Disorder, type unspecified [9]. Tics lasting for over a year are classified as Chronic Motor or Vocal Tic Disorders. Tourette Syndrome (TS) requires the presence of both motor and or vocal tics for at least one year. In general, the prevalence rates of TDs can be conceptualized as a pyramid in which transient, early onset tics serve as the base and TS represent the apex [9],[11].

**Table 1.** Tic disorder DSM-5 diagnostic criteria [9].

Criteria	Tourette's disorder 307.23 (F95.2)	Persistent (Chronic) motor vocal tic disorders 307.22 (F95.1) Specifiers: presence of motor or vocal tics	Provisional Transient tic disorder 307.21 (F95.0)	Other Specified Tic disorder 307.20 (F95.8) and Unspecified Tic Disorders 307.20 (F95.9)
Tics	Both motor and phonic tics	Motor or vocal tics	Motor or phonic	Motor or Phonic
Age of Onset	<18	<18	<18	Specify if >18
Duration	>1 year	>1 year	>4 weeks <1 year	<4weeks
Frequency	Multiple tics per day	Multiple tics per day	No pattern required	No pattern required
Exclusions	Drug effects- stimulants General medical conditions	Drug effects–stimulants General medical conditions	Drug effects- stimulants General medical conditions	Fails to meet criteria for other tic disorders

## 3. Differential diagnosis

There are several important steps in the differential diagnosis of TDs that provide useful clues about the genetic and pathophysiological origins of tics. The first involves differentiating primary from secondary tic disorders. This process centers on the exclusion of abnormal movements associated with clearly defined genetic, metabolic and neurodegenerative movement disorders. The second step involves differentiating tics from other sources of stereotypic movements, tardive and paroxysmal dyskinesias, myoclonus, substance and medication induced and abnormal movements associated with brain trauma [4]. The third step focuses on ruling out other sources of waxing-waning symptoms (mood and anxiety disorders), intrusive sensory and/or compulsive urges (e.g., akathisia, seizure disorders or PTSD); echo-phenomena, coprolalia, sensory and experiential gating disturbances, motor or cognitive perseveration and intrusive thoughts ideas, images and sensory experiences. The complex differential diagnosis of TD/TS should remind us that the multiple phenotypes of tics and other movement disorders are final common pathways for a variety of neurological, neuro-immunological, neurodegenerative, genetic/metabolic and neuropsychiatric disorders [12]–[14].

## 4. Tourette's syndrome

TS frequently presents a more complex clinical picture than the other Tic Disorders. These differences are in large part due to greater diversity and more complicated developmental trajectories during childhood and adolescence [15],[16]. Some of these differences reflect more than tic severity; they include individualized patterns of changing typology and topography. Initially most simple tics appear as involuntary movements that can be partially suppressed, wax and wane over time, worsen with stress or excitement, and improve or disappear during sleep. Many of these features help differentiate TS from other hyperkinetic movement disorders [10],[11]. As a result, the diagnosis of TS is usually one of exclusion even though the medical workup for most patients with mild TS is frequently negative.

As stated above, most affected preschool children present with transient tics. Yet embedded in this early onset group are children who will go on to develop chronic motor or vocal tics over the next 2–3 years. A subset of that group will begin with this initial sequence but go on to develop a combination of chronic vocal and motor tics characteristic of TS. Patients with either pattern of tics reach a peak of intensity within about five years of onset. The majority will experience a gradual waning and eventual disappearance of motor and vocal tics by late adolescence or early adulthood. Many of these adults can experience brief but transient resurgences during periods of increased stress [12],[13]. There is a subgroup of these patients with persistent TS. This cohort shares greater family loading (the presence of either OCD or tic disorders in both parental lineages); prenatal exposure syndromes (e.g., maternal smoking and other exposures to substances); maternal hyperemesis during pregnancy; history of intrauterine growth restriction; perinatal and early developmental insults (infections, trauma, and stress); and higher rates of co-occurring neurodevelopmental and psychiatric disorders [15],[17].

Qualitative changes in the complexity of tics and vocalizations also occur among individuals with severe or persistent TS. Complex tics are characterized by repetitive sequences of movements and vocalizations that are usually associated with sensory phenomena (sensations that precede tics) and/or sudden intrusive images and/or urges to complete these repetitive, ritualized, patterns of behaviors. If ignored, these urges and sensations will intensify, terminating only when the “voluntary” actions are completed or “feels right.” This combination of intrinsically motivated, “voluntary” movements and behaviors also resembles complex stereotypies, ritualized behaviors, compulsions without obsessions and addiction-like behaviors. In severe TS, echo-phenomena (repetitious motor behaviors) and aberrant social behaviors (touching genitals, coprolalia/copropraxia) can also cause significant psychosocial distress [11],[12],[16].

The population of patients with severe TS represents a very small fraction of those children with TD. At present, we have a limited understanding of how and why these changes occur. Clinically many of these patients are also unresponsive to standard treatments. Many resemble patients with ASD, addictions and OC spectrum or related disorders. In addition, many patients with sensory or premonitory tics, mixed sensorimotor abnormalities, or intrusive experiences blur the boundaries with other psychiatric disorders such as PTSD. Others may be misdiagnosed with bipolar disorder (waxing and waning symptoms); psychoses such as schizophrenia (intrusive images, thoughts and odd behaviors) and catatonia (negative tics, behavioral arrests, and odd posturing). This misattribution is most commonly associated with co-occurring ID or ASD [18].

Other repetitive behaviors include destructive and self-injurious behaviors that also possess addiction-like properties. These patterns of repetitive behaviors suggest an expanded role for the ventral cortico-striato thalamic motivational circuitry involved in other habit-related behaviors [19],[20]. Many of these the features are not routinely observed in most non-degenerative movement disorders [2],[10],[12],[21]. The evolution of these atypical features suggests a more complicated pattern of gene-environmental interactions. The best explanation combines a mixture of polygenic influences and a second hit or trigger stimulus that switches on during the course of the tic disorder. Although speculative, this pattern of progression resembles late neuroplastic changes that are analogous to kindling in recurring mood disorders; the development of mirror foci in chronic epilepsies; sensitization in the development of severe addictions, or perhaps the other disorders associated with repetitive neuronal activity [8],[16],[20],[22].

## 5. The problem of metamorphosis

Tic disorders vary in topography, typology and severity. These changes occur at three levels. The first involves waxing and waning course in which severity changes over time. The second is more likely associated with Tourette Disorder and involves the changing distribution and subtypes of motor and vocal tics. The initial clinical presentation of simple tics involves motor (eye blinking or grimacing) or vocal tics (sniffing and throat clearing). In TS, the pattern of emerging tics characteristically progresses from motor to vocal tics (or vice versa) within a year or so of onset although many patients deviate from this sequence. In most cases, the typologies of both motor and phonic tics will change over time [13],[15]. The third set of changes is more qualitative in nature. It is this group of tics that undergo significant changes in patterns of comorbidity as well as co-occurring behavioral (other repetitive behaviors, compulsions without obsessions, and self-injury); emotional (explosive emotional outbursts, impulsivity, mood/anxiety related symptoms); echo-phenomena, sleep disruptions, migraines, and other neurophysiological symptoms.

This group is characterized by greater genetic loading (especially in first-degree relatives in both maternal and paternal lineages), and is characterized by early onset tics, maternal smoking and co-occurring neurodevelopmental disorders. The transformation from simple tics is analogous to metamorphoses from one disorder into other far more complex neuropsychiatric conditions. Unfortunately, we know little about this process other than the likely role of neuronal antibodies, inflammatory activation, cumulative developmental insults, and epigenetic changes in this transformation. Expanding our knowledge base of these “malignant” forms of TS may require us to address tic disorders in terms of co-occurring neuropsychiatric disorders such as other genetic forms of movement disorder, and subtypes of OCRD and ASD [11],[16].

Previously we alluded to the artificial dichotomy between voluntary and involuntary movements. Simple tics are involuntary. Support for their involuntary nature come from neurophysiological examination of the premotor cortex and striatum. These studies demonstrate a lack of premotor potentials (contingent negative variation, CNV) that are associated with planned, voluntary movements. The absence of these automatic action potentials may explain why patents with simple motor tics do not appear goal-directed, serve any apparent communicative needs, and frequently go unnoticed [12],[14],[15],[23],[24].

Studies using CNV suggest a lack of readiness or pre-movement potentials preceding simple tics that changes with the emergence of sensory or premonitory tics. As described above, the urges intensify and drive what appear to be complex, voluntary movements. Transcranial magnetic stimulation (TMS) reveals a decrease in the Cortical Silent Period, and the decreased Short Interval Intra-Cortical Inhibition Responses. These changes are consistent with increased “arousal”, increased readiness and a lower threshold for sensorimotor activity. The mechanism seems most consistent with a deficit in baseline inhibition and top down regulation that are most likely related to decreased numbers of parvalbumin containing GABA interneurons that help regulate neuronal activity. Tic suppression represents a compensatory override of this system from the prefrontal cortex but unfortunately does not turn off the rising need to move [11],[25]. In this sense tic disorders resemble other psychophysiological or psychosomatic disorders [10].

Complex tics are preceded by sensory or premonitory sensations that induce the urge to tic. This pattern of behavior can be misattributed to drug-induced akathisia; craving-like behaviors seen in drug abuse; compulsive behaviors observed in OC-related disorders (non-anxious forms of OCD without obsessions), and in some treatment-resistant stereotypies/rituals [18],[26],[27]. Neurophysiologically these complex tics are preceded by pre-movement potentials best observed in the premotor and supplemental motor cortex. This same pattern of activation occurs prior to planned or practiced movements and suggests a more widespread, integrated pattern of neural activity. A second feature of complex tics involves the need to repeat until they “feel right”. This terminating event differs from most compulsions and repetitive movements that represent avoidance of or escape from anxiety or discomfort (OCD). They may evolve into patterns of automatic, approach behaviors that are relatively resistant to extinction and as such resemble habits and other addiction-like behaviors [2].

Patients with severe TD can vary widely in terms of social, emotional and psychological distress. In these cases, there is an apparent expansion of aberrant neurophysiological activity. For example, patients with tics and classic obsessive-compulsive disorder (anxiety type) present with a different pattern of TMS response that suggests a wider network [27],[28]. Functional neuroimaging suggests greater involvement/activity of prefrontal, caudate and limbic networks in the presence of obsessions and anxiety/avoidance behaviors [11],[15],[28],[29]. The pattern of activity deviates from the usual reciprocating relationships between frontal and parietal association cortices and subcortical/limbic networks [21],[25],[27],[30].

The polymorphic nature of complex tics frequently overshadows the recognition of tic disorders. In addition, their presence complicates the differential diagnosis by requiring a detailed investigation into other sources for sensory or prodromal experiences; OCD (with obsessions and anxiety) and repetitive, addiction-like behaviors symptoms; intrinsic sensory gating issues that give rise to intrusive visual or somatic experiences, and a range of imitative, or asocial ritualistic behaviors [11],[12],[27],[31]. On rare occasion the presence of negative tics (transient arrests in volitional movements) can be confused with on-off phenomena in Parkinson's disorder [16],[32],[33].

Co-occurrence of TD/TS with ID or ASD creates additional complications. For example, stereotypies, self-injury, ritualistic behaviors and impulsive, asocial behaviors create boundary issues with ASD and ID. These changes are not associated with specific gene markers but are surely driven by yet unknown gene-environmental/epigenetic forces. Like many convergence points, we can describe these far better than we can explain them [34]–[36]. In subsequent papers, we will explore these boundary issues, but for now, we can observe that these challenging behaviors provide a linkage between complex tics, abnormal movements associated with ID and the repetitive and restrictive behavior that are of diagnostic significance for ASD [18],[37],[38].

## 6. Role of psychosocial variables in TD

One major source of diversity and heterogeneity arises from chronic psychological, sexual or physical trauma during early childhood [39]. Chronic or repeated traumatization has an adverse effect on the developing stress-response system by derailing the typical entrainment and maturation of the hypothalamic pituitary axis, setting the balance between sympathetic/parasympathetic nervous systems, and the long-term organization and maturation of prefrontal cortical, striatal, limbic and thalamic circuitries. These disruptions can adversely affect developing executive and higher cortical functions that require hierarchically organized interconnections. This also disrupts the top-down regulation of subcortical circuitries, the emergence of sensory gating and regulation of attention, affect regulation, impulse control and developing sensorimotor networks [14],[40],[41].

On the surface, it appears that trauma provides a reasonable explanation for stress-related influences on tic disorders [42]. Current research suggests that trauma disrupts the development of several key neuronal circuits that affect the course of TD/TS. Changes in stress response and neuroendocrine networks contribute to impaired executive functions and sensory thresholds, anxiety or panic like events, intrusive emotional states, and anxiety/fear response regulation. Chronic trauma increases the risk for complex mood and anxiety disorders that contribute to increases in the frequency and intensity of tics and other movement disorders [11],[41]. In addition, these psychosocial insults occur in the context of genetic risk for tic disorders, possibly habit learning, and developing procedural and other types of memory and motor skills [20],[26]. These tic-related neurocognitive changes may affect not only the threshold or sensitivity to trauma but may also shape how those stressors are dealt with in the future [41],[43]. These issues should remind us of the complex, transactional nature of development and the multidirectional interaction between stress and underlying diatheses. Temperament, quality of attachment, learning experiences and individual and family psychosocial dynamics contribute to evolution of tic disorders. The sensitization of neuronal pathways by these experiences can alter the developmental trajectory of TS while also contributing to pattern of metamorphoses described throughout this article [6],[19],[43].

The patterns of aberrant development linked to ASD/ID, OCD, and TD are the result of altered neurogenesis, neuroplasticity, maturation of synaptic and axonal stability, and myelination. The impact of trauma-related events represents second or third hits in the emergence of complex TS as well as co-occurring behavioral and comorbid neuropsychiatric disorders [35],[37],[44]. The role of gender as a risk factor in these multidirectional stress-diathesis models is equally complicated. First, the majority of neurodevelopmental disorders display a strong gender bias towards males. However, when affected, many females can display more severe clinical phenotypes. In many studies, it appears that males have a lower threshold for expression that is offset by a greater vulnerability to the effects of “toxic” environments [27],[45],[46]. Even though females have lower prevalence rates, many require greater genetic loading for expression of the TS/TD, OCRD and ASD clinical phenotypes [15],[27]. In this sense, the stress-diathesis model suggests lower stress/greater genetic ratio for females when contrasted with males. Females on the other hand may be at greater risk for developing later onset trauma-related disorders or mood/anxiety disorders that may adversely affect quality of life and psychosocial adjustment [6],[41]. This complex pattern of gender dimorphism is illustrated by findings that suggest TS and OCD display a gender bias that is affected by age of onset; distribution of OCD and TD/TS in affected families; and the confusing relationship between genetic risk, gender, and severity of TS, OCD and OCD + TS. It remains to be seen whether these generalizations apply uniformly across the spectrum of TS + ASD and TS + ASD + OCD and how the presence of ID skews these observations [11],[18],[46].

## 7. Tics and other hyperkinetic movement disorders

TS is classified as a chronic, non-progressive hyperkinetic movement disorder that preserves characteristic traits like a waxing and waning clinical course, intensification during periods of excitement or stress, and lessening during sleep. The capacity to suppress abnormal movements observed in TD/TS is clear-cut from other hyperkinetic movement disorders [12],[31]. Tic disorders differ from other movement disorders in term of suggestibility (imitating the tics of others), the trend towards more complex symptomatology, and the emergence of sensory or prodromal tics [13],[32].

Movement disorders are also associated with mood disorders and neurocognitive changes observed in subcortical “dementias.” These neurocognitive changes include decreases in motivation, deficits in illicit or cued memory, dysfunctional sensory motor skills, sensory gating, set shifting and executive deficits related to motor speed, sequential skill learning, attention deficits (especially gating of intrusive motor activity) and adaptation of ongoing patterns of behavior. Embedded in these neurocognitive changes is a lower threshold for habit formation: a reliance on external cues to initiate behaviors (environmental dependency) and deficits in the automatization of practiced motor skills [45],[47],[48].

## 8. Genetic factors

TS is highly heritable (∼60%) [49]–[52]. First-degree relatives of TS cases have a 10–30 fold increased risk of TS compared with the general population, representing one of the highest recurrence risks in common neuropsychiatric diseases [53],[54]. But there are caveats. These studies reveal a significant heterogeneity among genetically related cohorts (e.g., monozygotic twins with Tourette's disorder). The concordance rate for monozygotic twins is less than 100% in TS in spite of the inclusion of all subtypes of tic disorders and OC Related disorders [27].

The search for specific gene markers in TD/TS began with Mendelian models of inheritance (autosomal dominance with variance penetrance). One variation of this model argued that a single gene was responsible for tics and associated conditions- ADHD, OCD and other disruptive behavioral disorders. Support for this model has waned greatly as a result of unsuccessful attempts to explain TD/TS in terms of specific chromosomal loci or candidate genes (e.g., genes encoding post-synaptic dopamine receptors, re-uptake transporter proteins, enzymes involved synthesis, vesicular dopamine release, and metabolism) [55],[56].

The genetic smoking guns remain elusive but there is evidence for a few rare, highly penetrant mutations (i.e., *HDC*, *SLITRK1*, and *CNTNAP2*) found in single families or a few cases [57]–[61]. Rare CNVs have been associated with TS (e.g., deletions in 22q11, *NRXN1*, *NLGN4*) [62]–[64], but the sample sizes for these association studies were small and replication is required. Exome sequencing has been applied to one TS pedigree [65] and larger trio studies are currently underway. As with other psychiatric disorders, the genetic architecture of TS is therefore complex, and large numbers of cases will be required to identify specific susceptibility genes. The first genome-wide association study (GWAS) of TS [66] sampled 1496 cases and 5249 controls but no genome-wide significant loci were identified. Polygenic risk analysis [67] indicated that increased sample sizes are likely to yield significant loci and that rare variants explain ∼20% of TS heritability.

Although gene-environment interactions (epigenetic factors) are involved, there is little definitive evidence for genomic imprinting as an explanation for gender dimorphism (males with tics; females with OCD). Other neurodevelopmental disorders display a similar pattern of gender dimorphism [8],[11],[68]. As noted above, the introduction of more precise molecular genetic tools has not yet yielded definitive gene markers. Currently available studies suggest significant heritability and the likelihood of polygenic inheritance. We are left to speculate about the underlying mechanisms for the progression of tic disorders. In all probability, epigenetic effects operate on multiple genes associated with an expansion of tic-related symptoms in multiple different brain areas based on neuroimaging studies of the cortico-striato-thalamic-cortical networks. Some of these changes represent altered intracellular mechanisms (second messengers, gene activation, nuclear transport and regulation of regional transcription). Recent data suggest a role of multiple neuroendocrine networks; activation of neuro-inflammatory pathways; multiple neurotransmitter systems; neuropeptides that influence motor behaviors and attachment related behaviors (oxytocin and vasopressin), and environmental/ecological forces [8],[69],[70].

## 9. Pathophysiology

TD/TS are complex phenotypes that represent a final common pathway within and between cortico-striato-thalamic-cortical (CSTC) networks. Early on, simple tics are basically involuntary and involve over excitable premotor and motor pathways. The emergence of premonitory or sensory tics broadens the scope of participating neuro-circuits and marks a shift to both involuntary and urge-driven voluntary movements [2],[45]. Complex tics represent a convergence of simple and mixed tics that also include echo-phenomena, suggestibility and a complex pattern of repetitive behaviors that are sometimes difficult to differentiate from compulsions. The neuro-circuitry expands to include association cortices, prefrontal and ventral medial circuits. At this stage, the interaction between motor and limbic systems leads to a more complex set of motivating forces that eventually shift from goal-directed to habitual modes of responding to both internal and external events [15],[18],[28].

Historically, hyperkinetic movement disorders were lumped into disorders associated with excessive postsynaptic dopamine activity (hyperdopaminergic state). Although dopaminergic dysregulation plays a central role, it is by no means the only neurotransmitter system involved in the neuropharmacology of TD/TS. Abnormal movements result from an imbalance between dopamine/cholinergic/GABAergic activities within CSTC networks. Unfortunately, even this revised model does not fully explain the sensitivity of most tic disorders to low dose neuroleptic drugs, the lower threshold for extrapyramidal symptoms (including akathisia) and the subsequent increased risk for tardive dyskinesia [16],[45],[71]. In spite of its utility for uncomplicated TD/TS, it falls short when dealing with complex tic disorders. For the small group of severely affected probands, we need to understand the factors related to the progression of simple tics to complex TS associated with neurocognitive and neurobehavioral complications. These changes suggest an expansion into other interlocking neuronal networks. In this model, multiple neurotransmitters, neuromodulators, inflammatory peptides/cytokines and neuro-hormones are operating at key nodal points that interconnect the limbic system, prefrontal regions devoted to executive functions and other cortical regions. These nodal points represent the intersection of brain networks devoted to emotional perception; behavioral responses to both internal and external stimuli; regulation of attention/impulse control; and motor initiation; adapted to social contexts and communication; motivational states, as well as error detection and self-appraisal [11],[45].

These caveats point to several crucial issues in this paper. As clinicians and researchers, it is important to think of TD/TS as syndromes with many subtypes and phenocopies. The same level of diversity applies to OCD and related disorders. For example, TD shares a genetic relationship with OCRD but is also associated with high rates of comorbidity with ADHD, anxiety and developmental learning disabilities. As a result, it was assumed that ADHD and TD/TS also shared common risk factors and therefore were genetically related syndromes. More recent research challenges this assumption. The evidence suggests that OCRD and TD/TS are interrelated but ADHD is not directly so. The relationship between ADHD and TD/TSD is more consistent with comorbid but independent syndromes except when ADHD is associated with OCRD. This study needs replication but does suggest that the only shared genetic risk between ADHD and TD/TS is related through the OCRD + ADHD connection [8],[27],[72].

Lacking a comprehensive explanation for the nearly 70% rates of co-occurrence between TD/TS and ADHD we turn to gender effects- the bias towards males for both disorders. The gender bias is observed in the majority of neurodevelopmental disorders but whether this relationship solves the problem remains to be seen. There is a second caveat. The combination of ADHD and TD/TS has an adverse effect on impulse control, levels of explosive and aggressive behaviors, irritability and emotional outbursts behaviors, and many asocial behaviors usually associated with TD/TS [11],[35],[73],[74].

## 10. Neurophysiology, Neuroanatomy and Metamorphosis

The complexity and integration of these cortico-striato-thalamic-cortical networks creates a “no man's land” for clinicians. Perhaps the biggest challenge involves developing an understanding of the functional neuroanatomy that leads to the typological, typographical and changing severity of tics. This gap widens when the focus expands to include the forces behind symptom changes over time and makes it difficult to predict the developmental trajectory of tic disorders over time. For those with severe TD/TS there is a limited understanding of the basic functional neuroanatomical changes that contribute to the emergence of challenging behaviors (stereotypies, aggression, repetitive behaviors, SIB and in some circumstances, catatonia), sensorimotor and neurocognitive symptoms and comorbid mental disorders [16],[19],[31],[75].

Tic disorders represent a complex genetic disorder with heterogeneous clinical phenotypes. The level of heritability in part varies with age of onset, gender, comorbidity with ADHD and OCRD, and relationship to neurological, immunological, neurodevelopmental and other movement disorders [2],[8],[12]. The boundaries between tic and OCRD is the most difficult to clearly define. For example, the highest concordance ratios are between tics and OCRD. For individuals with Tourette's Disorder, OCRD is present in nearly 40% of patients. This group seems to have the highest genetic loading and more a severe form of both disorders [15],[16],[27],[45]. As noted earlier, families with extensive pedigrees for TD/TS may also have more female relatives with OCRD. Early onset OCD may also reflect a greater level of genetic loading, but as many as 50% of affected preschool males will develop tics within 5 years [14],[19]. Others with early onset tics may develop more persistent stereotypies, compulsions, impulsive addiction-like behaviors, and complex tics frequently preceded by sensory tics or urges. These vulnerabilities suggest that an early age of onset may have more complex effects on brain development and subsequent clinical symptoms [72].

## 11. Conclusions

This paper addresses the complex neurobiology of TD/TS and provides a glimpse into a family of comorbid and co-occurring neuropsychiatric disorders. It outlines some of the areas in which descriptive/categorical diagnostic criteria are less helpful and emphasize the increasing need to define and refine neurobiological endophenotypes. Subsequent articles will explore the many relationships between OCRD, ASD and TD/TD. One primary focus is on each syndrome as a complex genetic disorder that share overlapping functional neuroanatomy, neuropharmacology and clinical symptomatology.

The greatest barrier to TD/TS research lies in its diversity and heterogeneity. This variability arises from the large number of neurological, genetic and medical phenocopies. These problems reinforce our understanding of TS/TD as one pattern of phenotypic expression that must be considered in the context of more generalized sensorimotor disinhibition, imbalance between excitatory/inhibitory networks and cortical dysregulation. Much of the variability encountered by clinical and researchers requires us to consider TS/TD as the expression of multiple independent but co-occurring disorders; the product of polygenic inheritance whose phenotypic expression varies in relation to the particular mixture of genes and gene-environment interactions, and the effects of gender on phenotypic expression [8],[16],[44],[45],[69],[76].

One approach to address these issues was provided by NIMH in 2013- the Research Domain Criteria [77]. This methodology incorporates psychobiological features of attachment behaviors, temperamental, neuro-endocrine, neurophysiological and neuropharmacological elements into classifying behavioral traits. When applied to complex neurodevelopmental disorders, this approach allows the clinician to move beyond co-occurring descriptive syndromes and into the neurobiological traits the may serve to link them [12],[16],[19].
